# A Fast and Efficient Method for Radiation Pattern Prediction in Large-Scale Tightly Coupled Linear Antenna Arrays

**DOI:** 10.3390/s26092795

**Published:** 2026-04-30

**Authors:** Jianshu Wei, Peng Xu, Haitao Lu, Xiao Cai

**Affiliations:** 1 Research Center of Applied Electromagnetics, Nanjing University of Information Science and Technology, Nanjing 210044, China; 202312180020@nuist.edu.cn (J.W.); 202511180007@nuist.edu.cn (H.L.); 2 School of Information Engineering, Jiangsu Maritime Institute, Nanjing 211199, China; xup2016@126.com; 3 Nanjing Huihai Transportation Technology Co., Ltd., Nanjing 210036, China

**Keywords:** beamforming, fast simulation, large-scale antenna arrays, radiation pattern prediction, tightly coupled arrays

## Abstract

Reliable and fast radiation pattern prediction is critical for large-scale tightly coupled linear antenna arrays. Strong mutual coupling and finite-array edge effects limit the accuracy of conventional array factor methods, while full-wave simulations become computationally prohibitive for large arrays. To address this issue, a fast and efficient radiation pattern prediction method (FERPP) is proposed. For central elements, the far-field response is obtained from a calibrated reference array and extended through position-dependent phase compensation. For edge elements, responses are extracted from independent local full-wave simulations. All element responses are assembled into a global far-field response matrix, enabling direct radiation pattern synthesis using the extended method of maximum power transmission efficiency. Simulation results obtained with a 1024-element linear microstrip patch antenna array operating at 3.5 GHz, with small inter-element spacing, demonstrate close agreement with full-wave simulations. For a broadside single-beam case, the predicted peak gain is 29.10 dBi, compared with 29.02 dBi from full-wave simulation. For a scanned beam at 30°, the predicted peak gain is 28.22 dBi, while the full-wave result is 28.99 dBi. For an equal-weight three-beam configuration at −30°, 0°, and 30°, the proposed method yields a peak gain of 23.87 dBi, compared with 24.21 dBi from full-wave simulation. In terms of computational efficiency, the proposed method requires only about 1.8% of the computational time required for a full-wave simulation. These results demonstrate that the proposed FERPP method provides a practical and efficient solution for radiation pattern prediction and beamforming analysis of large-scale tightly coupled linear antenna arrays.

## 1. Introduction

Large-scale antenna arrays play an essential role in modern wireless communication, radar, and sensing systems, as they provide high gain, flexible beam control, and spatial selectivity. The theoretical foundation of phased array antennas has been well established in classical literature, where the principles of array synthesis and beam steering were systematically formulated [1]. With the rapid development of large-scale and extremely large-scale antenna systems, reliable and efficient modeling and radiation pattern prediction have become increasingly important, particularly in massive MIMO and future wireless networks [2].

In practical engineering applications, phased array antennas are widely employed in millimeter-wave communication systems and spaceborne platforms. Representative examples include switched-beam antennas for 5G millimeter-wave applications [3], high-efficiency millimeter-wave phased arrays [4], and low-profile beamforming arrays for radar systems [5]. Active phased arrays are also extensively used in satellite payloads and space-based communication platforms, where high reliability and flexible beam control are required [6,7,8]. In many of these designs, compact element spacing is adopted to meet size constraints and improve integration density.

When the inter-element spacing becomes electrically small, electromagnetic interactions among array elements are significantly enhanced. In finite arrays, edge elements experience boundary conditions that differ from those of interior elements, leading to pronounced edge effects that strongly influence radiation characteristics [9]. This problem is further intensified in tightly coupled array configurations, where mutual coupling cannot be neglected and conventional impedance matching techniques may become ineffective [10]. Under such conditions, reliable radiation pattern prediction becomes a challenging task in array design.

To efficiently evaluate antenna array radiation characteristics, several analytical approaches have been developed, among which the array factor method is widely used due to its simplicity and low computational cost [11]. By modeling antenna elements as ideal point sources, it enables fast pattern estimation and works well for arrays with moderate electrical size and weak coupling. For large finite arrays with compact element spacing, however, strong mutual coupling and edge effects invalidate the point-source assumption. Since these effects are not included in conventional array factor formulations, noticeable discrepancies may arise when compared with full-wave simulations or measurements, especially for tightly coupled arrays [12,13].

From a numerical standpoint, full-wave electromagnetic simulation provides a rigorous description of array behavior by directly accounting for mutual coupling and boundary effects. Early studies demonstrated fast full-wave analysis techniques for large finite arrays of microstrip antennas [14], and subsequent advances in numerical acceleration methods, such as multilevel fast multipole algorithms and domain decomposition techniques, have further extended the capability of large-scale simulations [15,16]. Nevertheless, for arrays with hundreds or thousands of elements, the computational cost remains prohibitively high, particularly when repeated simulations are required during design optimization or excitation adjustment. Similar challenges have also been reported in the analysis of scattering and radiation from electrically large arrays [17,18].

To balance modeling accuracy and computational efficiency, active element pattern (AEP)–based methods have been widely adopted as an intermediate approach between simplified analytical models and full-wave simulation. The active element pattern concept was introduced to account for mutual coupling effects by embedding each element in its actual electromagnetic environment [19]. Subsequent studies examined its behavior in finite arrays and demonstrated its effectiveness for coupling-aware radiation pattern prediction [20]. More recent works have proposed efficient techniques for estimating active element patterns in planar arrays, enabling faster evaluation of array radiation characteristics [21,22]. Coupling-aware fast synthesis and evaluation methods have also been developed to further improve efficiency while maintaining acceptable accuracy [23,24,25].

Despite these advantages, conventional AEP-based approaches are usually built on the assumption that all array elements experience a similar electromagnetic environment, which is typically represented by a single reference element. This assumption becomes less accurate in large finite arrays, where edge elements operate under boundary conditions that differ significantly from those of central elements. To better represent spatial nonuniformity in large arrays, region-based modeling approaches based on irregular subarray tiling have been explored [26]. In addition, related beam synthesis and pattern-shaping techniques have also been developed for multibeam and shaped-beam array systems [27,28]. To further improve prediction performance, partition-based AEP methods have been proposed, in which the array is divided into different regions and representative active element patterns are assigned according to the local electromagnetic environment [29,30]. Although these approaches outperform single-reference AEP models, the choice of partition schemes and representative elements is often empirical, and accurate pattern extraction may still require full-array simulations or carefully designed subarrays [31]. As a result, achieving a favorable balance between prediction fidelity and computational efficiency remains challenging for large-scale tightly coupled arrays.

Based on these considerations, this work focuses on reliable and efficient radiation pattern prediction for large-scale tightly coupled linear antenna arrays. The proposed method is related to existing AEP-based and partition-based modeling ideas in that it also exploits the spatial nonuniformity of the electromagnetic environment in finite arrays. However, its distinction does not lie only in the determination of the boundary between the central and edge regions. More importantly, the boundary size is selected through a convergence-driven calibration procedure, the central-region responses are constructed from a calibrated reference response obtained from a compact calibration array, and the edge-region responses are explicitly established through element-wise local full-wave simulations. In this way, a position-dependent prediction framework is formed for large-scale tightly coupled linear arrays. By combining compact full-wave simulations with calibrated field extraction, the proposed method achieves close agreement with full-wave simulation results while significantly reducing computational cost. Therefore, the proposed method provides a systematic and physically consistent solution for radiation pattern prediction and beamforming analysis of large-scale tightly coupled linear antenna arrays.

## 2. Fast and Efficient Radiation Pattern Prediction Method

### 2.1. Array Partition Strategy and Calibration Array Construction

Consider a finite large-scale linear antenna array with *m* radiating elements. In such arrays, elements located sufficiently far from the physical boundaries experience an approximately periodic electromagnetic environment, whereas elements located near the array boundaries are more strongly affected by position-dependent boundary effects [12,13]. Therefore, the electromagnetic response of each element depends on its position within the array, particularly in regions where boundary effects break the periodic electromagnetic environment.

**Figure 1:**
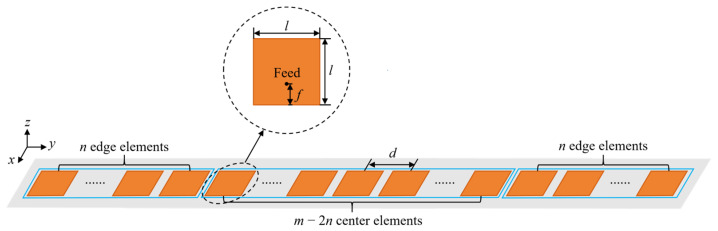
Partitioned modeling of the antenna array.

Based on this observation, a boundary region is defined to include the elements located within *n* elements from each end of the array. The value of *n* is determined through a convergence-based calibration procedure under the given operating frequency and element spacing, such that coupling effects beyond this range become sufficiently small. Accordingly, the array is divided into two regions with distinct electromagnetic characteristics: the central region, containing the middle *m* − 2*n* elements, and the edge region, containing the 2*n* boundary elements, as illustrated in Figure 1. In the central region, the electromagnetic environment experienced by the elements is approximately periodic due to the relatively large distance from the physical boundaries. Therefore, within the selected central region, the responses of these elements can be approximately represented by a calibrated central-element model. In contrast, elements located in the edge region are directly influenced by array truncation and boundary-induced coupling variations, which must be explicitly considered in the modeling process.

To model the central region consistently, a calibration array is constructed using the same element geometry, materials, and spatial arrangement as the original array. The calibration array contains 2*n* + 1 elements, which represents the minimum effective size required to reproduce the electromagnetic environment experienced by a central element in a large finite array. The choice of this calibration size directly affects the quality of the predicted element response and the computational cost.

An iterative procedure is used to determine the optimal value of *n*. Starting from an initial value of *n* = 0, a calibration array with 2*n* + 1 elements is simulated. The realized gain pattern of the central element along the H-plane is then extracted and denoted by $G_{n}(\theta )$. The root-mean-square (RMS) error between two successive iterations is evaluated over the sampled H-plane angular region as

(1) $$a_{n}=\sqrt{\frac{1}{181}\sum_{\theta =-90{}^{\circ}}^{90{}^{\circ}}{\left(\left|G_{n}(\theta )\right|-\left|G_{n-1}(\theta )\right|\right)}^{2}}.$$

$G_{n}(\theta )$ and $G_{n-1}(\theta )$ denote the realized gain patterns of the central element obtained from calibration arrays with sizes *n* and *n* − 1, respectively. The summation is carried out over the sampled H-plane angular region, −90°≤θ≤90°. If $a_{n}\leq \delta$, where δ is a predefined convergence threshold, the electromagnetic response of the central element is considered converged, and the corresponding *n* is selected as the minimum effective boundary size.

This convergence-based strategy ensures that the calibration array is neither oversized nor insufficient for representing the central electromagnetic environment.

### 2.2. Central-Region Equivalent Modeling and Edge-Region Accurate Simulation

**Figure 2:**
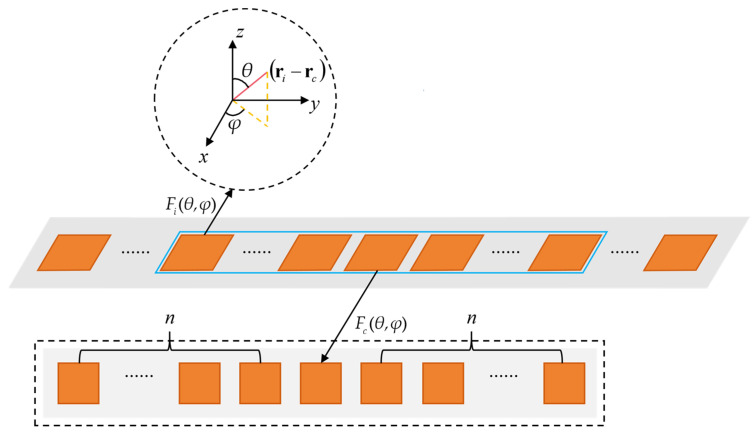
Calibration array and central element response extraction.

After the calibration array size has been determined, a full-wave simulation of the calibration array is performed to extract the far-field response of the central element, denoted as $F_{c}(\theta ,\varphi )$, as shown in Figure 2, where the dashed box indicates the corresponding calibration array. This response represents the radiation behavior of a typical central-region element under realistic coupling conditions and is consistent with the active-element-pattern concept [19,20].

This extracted response serves as the reference for the subsequent equivalent modeling of the selected central region. Since the corresponding element is located at the center of the calibration array, its electromagnetic environment is most representative of the approximately periodic interior region of the finite array. Therefore, the calibrated central-element response provides a physically meaningful basis for extending the response to other central-region elements through position-dependent phase compensation.

For elements located in the selected central region, their far-field responses are approximated from the calibrated central-element response through spatial phase translation and are assembled into the central-region response vector as

(2) $$\begin{array}{l} {}[F_{\mathrm{center}}(\theta ,\varphi )]=[F_{n+1}(\theta ,\varphi ),\;F_{n+2}(\theta ,\varphi ),\ldots ,\;F_{m-n}(\theta ,\varphi )],\;\\ F_{i}(\theta ,\varphi )\approx F_{c}(\theta ,\varphi )e^{-jk(r_{i}-r_{c})\cdot \hat{r}(\theta ,\varphi )},\;i=n+1,\ldots ,\;m-n. \end{array}$$

Here $F_{c}(\theta ,\varphi )$ denotes the calibrated far-field response of the central element, *k* = 2π/λ is the free-space wave number, $r_{i}$ and $r_{c}$ denote the position vectors of the *i*-th element and the central element, respectively, and $\hat{r}(\theta ,\varphi )$ is the unit vector along the observation direction. The exponential term accounts for the phase shift caused by spatial displacement.

Based on (2), the far-field response of each element within the selected central region is approximated by the reference response of the calibrated central element together with a position-dependent phase shift. This approximation does not imply that the interior elements are strictly identical apart from phase shift. Rather, it is applied only within the selected central region determined by the convergence-based calibration procedure, where the residual variation of the element responses remains sufficiently small for the present prediction task. The edge and near-edge elements are still modeled explicitly.

**Figure 3:**
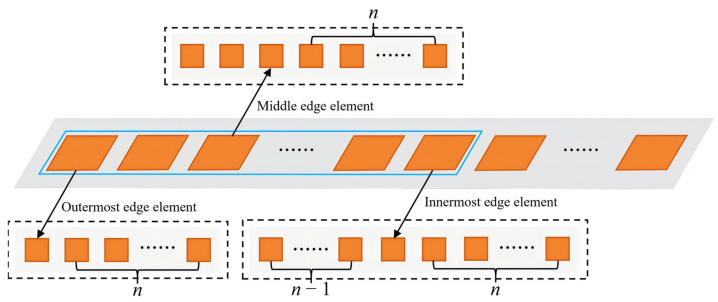
Compact subarrays for edge element simulations.

Since the approximately periodic electromagnetic environment does not hold near the boundaries, edge elements require independent treatment. As illustrated in Figure 3, each edge element is modeled using an independent local full-wave simulation based on a compact subarray that includes the element of interest and its nearest neighbors.

This localized modeling strategy follows existing approaches for large arrays [21,22,23], while accounting for the position-dependent electromagnetic environment of each edge element. The far-field responses of the edge elements are assembled as

(3) $$[F_{\mathrm{edge}}(\theta ,\varphi )]=[F_{1}(\theta ,\varphi ),\ldots ,\;F_{n}(\theta ,\varphi ),\;F_{m-n+1}(\theta ,\varphi ),\ldots ,\;F_{m}(\theta ,\varphi )],\;$$

where $F_{i}(\theta ,\varphi )$ denotes the far-field response of the *i*-th edge element obtained from the corresponding local full-wave simulation. In contrast to the central region, where the responses of interior elements can be approximated through spatial phase translation, the edge-region response must be established explicitly in order to preserve the position-dependent coupling variations induced by truncation and boundary effects. By combining equivalent modeling for central elements with accurate simulation for edge elements, the position-dependent nature of coupling effects can be effectively captured across the entire finite array.

### 2.3. Global Field Integration and Beamforming Optimization

After obtaining the accurate or equivalent far-field responses of all array elements, the responses of the central and edge regions are assembled according to the physical ordering of the array to form the global response of the array. Specifically, the global response is constructed by combining $\left[F_{\mathrm{center}}(\theta ,\varphi )\right]$ and $\left[F_{\mathrm{edge}}(\theta ,\varphi )\right]$ according to the actual element positions in the array. For beam synthesis, a finite set of prescribed angular positions $\left\{\left(\theta_{1},\varphi_{1}\right),\;\left(\theta_{2},\varphi_{2}\right),\ldots ,\;\left(\theta_{x},\varphi_{x}\right)\right\}$ is specified according to the desired beam configuration and selected within the prescribed observation region. By extracting the corresponding directional response samples at these angular positions, a response matrix is constructed for subsequent excitation optimization.

To synthesize the desired radiation pattern, the optimal excitation vector $\left[w_{opt}\right]$ is determined based on the extended method of maximum power transmission efficiency (EMMPTE) [27,28], which provides a physically meaningful criterion for array excitation design under coupled electromagnetic environments. The detailed implementation procedure is not repeated here for brevity and can be found in [32]. The resulting optimal excitation vector $\left[w_{opt}\right]$ is then used for subsequent radiation-pattern evaluation.

Based on the synthesized array response, the final radiation performance is evaluated in terms of realized gain as

(4) $$G_{\mathrm{realized}}(\theta ,\varphi )=20\log_{10}\left(\frac{\left[F(\theta ,\varphi )][w_{opt}\right]}{F_{0}}\right),$$

where [F(θ,φ)] denotes the synthesized global far-field response of the array in the observation direction (θ,φ), and $F_{0}$ is the reference response magnitude corresponding to a normalized input power. This formulation ensures that the synthesized array response is evaluated consistently in terms of realized gain under practical port-matching conditions.

Overall, the proposed FERPP method combines convergence-based calibration, regional response modeling, and beam synthesis into a unified prediction procedure. The central-region responses are constructed from a calibrated reference response through position-dependent phase compensation, whereas the edge-region responses are established by independent local full-wave simulations. These responses are then assembled into the global far-field response of the array for subsequent excitation determination and realized-gain evaluation.

## 3. Results and Analysis

### 3.1. Simulation Setup

To evaluate the practical performance of the proposed FERPP method, a 1024-element linear microstrip patch antenna array operating at 3.5 GHz is selected as the validation platform, as illustrated in Figure 1. The patch element is optimized with dimensions of *l* = 19.7 mm, *f* = 5 mm. The antenna array has a uniform inter-element spacing of *d* = 0.245*λ*_0_, where *λ*_0_ denotes the free-space wavelength, and is printed on the FR4 substrate with a thickness of 2 mm, relative permittivity of 4.4, and loss tangent of 0.02.

All electromagnetic simulations are conducted on the same computer platform equipped with an Intel Core i5-12400 processor, an NVIDIA GeForce RTX 3060 Ti graphics card, and 64 GB of system memory, ensuring the consistency and reliability of the numerical results reported in this work.

### 3.2. Determination of the Calibration Array Size and Model Validation

Determining the minimum effective size of the calibration array *n* is essential for achieving accurate and efficient equivalent modeling of the central region. To this end, a dynamic convergence evaluation strategy based on the response stability of the central element is employed to validate the rationality of the calibration array size.

**Figure 4:**
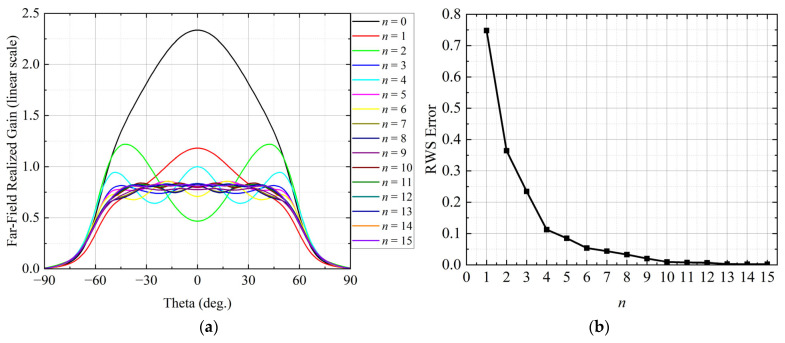
Convergence of the calibration array size. (**a**) H-plane realized-gain patterns of the central element in linear scale for different numbers of neighboring elements *n*. (**b**) RMS error versus *n*.

As illustrated in Figure 4a, calibration arrays with the number of neighboring elements *n* ranging from 0 to 15 are successively simulated. For each calibration array, the H-plane far-field realized gain of the central element is extracted. For the considered linear array, the H-plane is adopted in the convergence evaluation because it most directly reflects the main-beam evolution, steering direction, and sidelobe characteristics, and therefore provides a relevant basis for assessing the convergence behavior of the central-region modeling. The convergence behavior is quantitatively evaluated by computing the root-mean-square (RMS) error $a_{n}$ between the realized-gain curves obtained from two successive calibration arrays with sizes *n* and *n* − 1 over the mainlobe region (−90°≤θ≤90°). The resulting convergence curve of *a_n_* as a function of *n* (1 ≤ *n* ≤ 15) is shown in Figure 4b.

As *n* increases from 1 to 7, the RMS error decreases rapidly from 0.75 to 0.04. When *n* = 7, the RMS error falls below the predefined convergence threshold of δ=0.05. As *n* continues to increase beyond this point, the reduction in RMS error becomes marginal and the convergence curve gradually approaches a stable plateau. Therefore, a 15-element calibration array, consisting of the central element and its seven nearest neighbors on each side, is sufficient to characterize the central electromagnetic environment in the considered large finite array with strong mutual coupling. This provides a reliable basis for the subsequent equivalent modeling of the central-region responses.

The threshold δ=0.05 is adopted as a practical criterion to balance modeling accuracy and computational cost. As indicated by the convergence trend in Figure 4b, the improvement beyond *n* = 7 is limited, while a looser threshold would reduce the calibration size at the expense of modeling fidelity.

**Figure 5:**
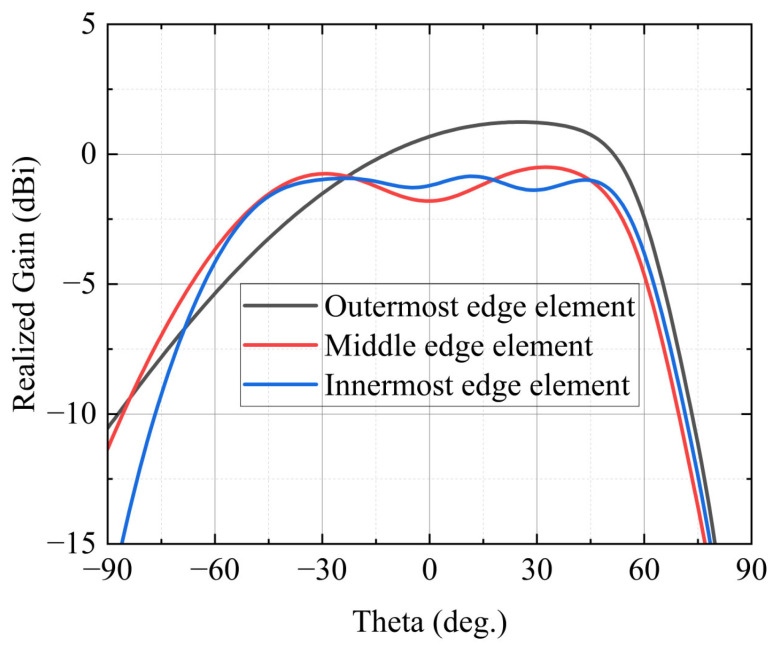
H-plane realized-gain patterns of representative edge elements.

In addition to determining an effective calibration-array size for the central region, the applicability of the proposed strategy to edge elements is examined using compact subarray simulations with *n* = 7. Figure 5 shows H-plane realized-gain patterns of three representative edge elements, namely, the outermost edge element (element 1), the middle edge element (element 3), and the innermost edge element (element 7), whose positions along the array are indicated in Figure 3.

Although all three elements belong to the edge region, their radiation characteristics vary markedly, as shown in Figure 5. The outermost edge element (black curve) exhibits the most severe pattern distortion, with its main lobe clearly tilted away from broadside and peaking around 30°. The middle edge element (red curve) exhibits less severe distortion, with its main lobe shifted closer to broadside while still maintaining a distinct sidelobe structure. In contrast, the innermost edge element (blue curve) exhibits a radiation pattern more similar to that of a central element, with a relatively stable radiation response over a wide angular range from approximately −30° to 50°, along with moderate ripple and asymmetry.

These observations demonstrate the position-dependent nature of edge effects in finite arrays. As the element location moves from the physical boundary toward the array interior, the influence of boundary-induced coupling gradually weakens, leading to a continuous evolution of the radiation response. Therefore, a uniform edge model cannot adequately capture these behaviors. To address this issue, a one-element–one-model simulation strategy is adopted for edge elements, so that the spatially varying electromagnetic environment near the array boundaries can be explicitly represented. By combining the equivalent modeling of central elements with the explicit modeling of edge elements, the proposed approach provides a more physically consistent representation of the finite-array environment and establishes a reliable basis for subsequent beamforming analysis.

## 4. Performance Comparison and Discussion

### 4.1. Comparison Methods

To evaluate the effectiveness of the proposed fast and efficient radiation pattern prediction method (FERPP), a systematic comparison is conducted with several representative prediction approaches for large-scale antenna arrays. All methods are applied to the same 1024-element tightly coupled linear array under the array configuration and excitation settings described in Section 3. Full-wave simulation results obtained using CST Microwave Studio 2020 are taken as the reference benchmark. Consistent with the methods reviewed in Section 1, four representative approaches are considered: the array factor method, the conventional AEP method, the partition-based AEP method, and the proposed FERPP method.

For the array factor method, all elements are assumed to have identical element patterns. For the conventional AEP method, the active element pattern of the central element is used to represent all array elements. For the partition-based AEP method, the array is divided into central and edge regions: the central-element active pattern is used for all interior elements, while representative edge-element patterns extracted from full-wave simulations of the corresponding edge subarrays (*n* = 7) are assigned to the edge region. For the proposed FERPP method, the central-element response is extracted from a calibration array and extended to other central elements through spatial phase translation, whereas edge-element responses are obtained from independent compact subarray simulations, following the modeling procedure described in Section 2.

**Figure 6:**
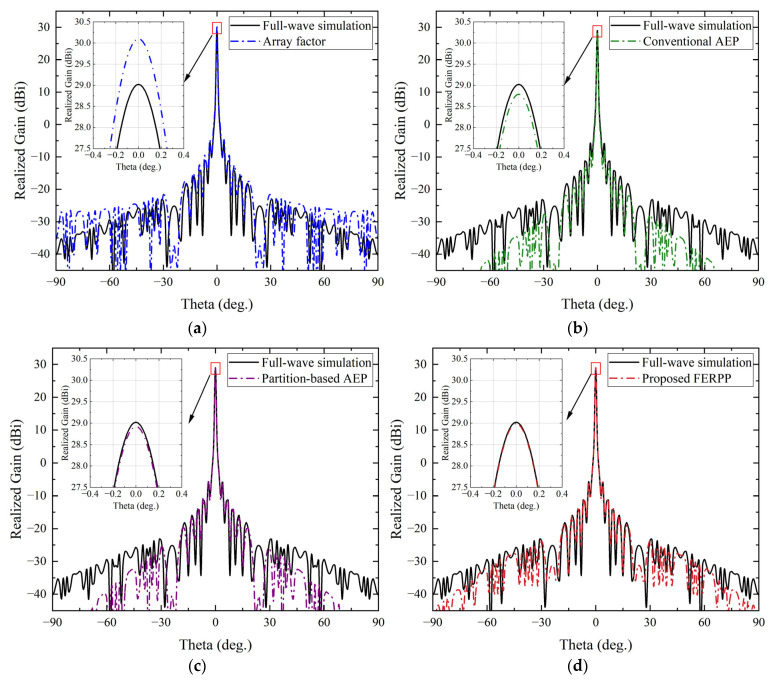
Radiation pattern comparison in the H-plane for the single-beam case at *θ* = 0°. (**a**) Comparison between full-wave simulation and array factor. (**b**) Comparison between full-wave simulation and conventional AEP. (**c**) Comparison between full-wave simulation and partition-based AEP. (**d**) Comparison between full-wave simulation and proposed FERPP.

**Figure 7:**
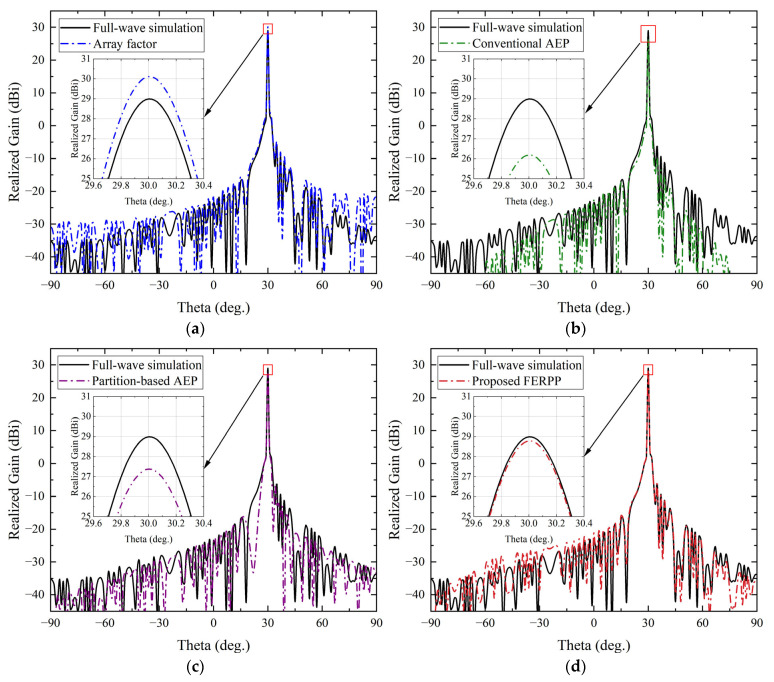
Radiation pattern comparison in the H-plane for the single-beam case at *θ* = 30°. (**a**) Comparison between full-wave simulation and array factor. (**b**) Comparison between full-wave simulation and conventional AEP. (**c**) Comparison between full-wave simulation and partition-based AEP. (**d**) Comparison between full-wave simulation and proposed FERPP.

**Figure 8:**
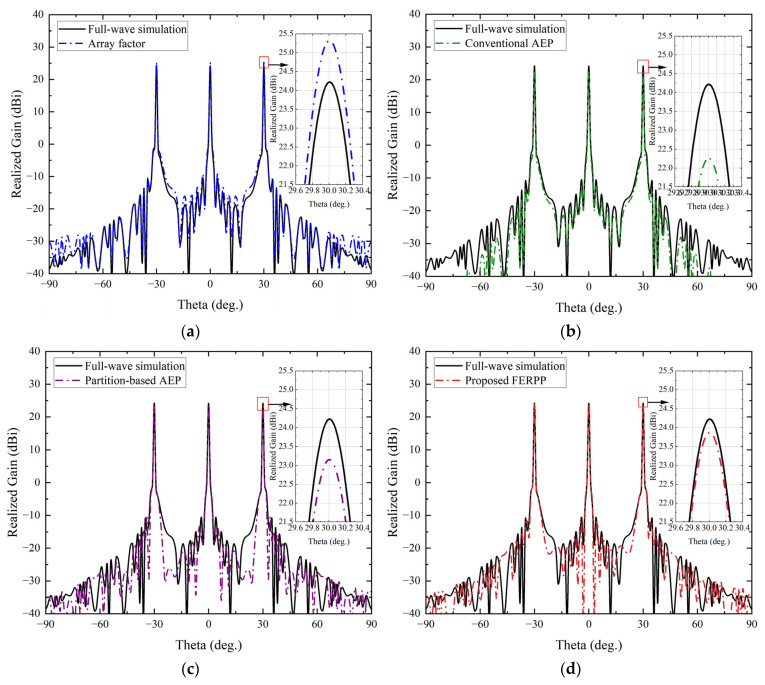
Radiation pattern comparison in the H-plane for the equal-weight three-beam configuration. (**a**) Comparison between full-wave simulation and array factor. (**b**) Comparison between full-wave simulation and conventional AEP. (**c**) Comparison between full-wave simulation and partition-based AEP. (**d**) Comparison between full-wave simulation and proposed FERPP.

To ensure a fair comparison, the same excitation vectors and input-power normalization are used for all methods. The evaluation focuses on the maximum realized gain within the prescribed beam-shaping region and the corresponding sidelobe level, both measured relative to the full-wave simulation results. The corresponding radiation patterns are shown in Figure 6, Figure 7 and Figure 8.

### 4.2. Beamforming Performance Under Different Scenarios

This section presents the numerical results under representative beamforming scenarios, with emphasis on the performance of the proposed FERPP method relative to full-wave simulation. The analysis considers the maximum realized gain within the prescribed beam-shaping region and the corresponding sidelobe level. In addition, the computational cost of each method is evaluated in terms of total simulation time and memory usage. Since the computational burden mainly depends on the prediction strategy and hardware configuration rather than the specific beamforming case, the computational cost remains largely consistent across different scenarios. The detailed results are presented in Section 4.2.1, Section 4.2.2 and Section 4.2.3.

#### 4.2.1. Broadside Single-Beam Radiation (*θ* = 0°)

Figure 6 shows the H-plane radiation patterns predicted by different methods for the broadside single-beam configuration. This case represents the simplest beamforming scenario and is therefore used to evaluate the fundamental radiation pattern prediction capability of the proposed FERPP method (*θ* = 0°). The comparison provides a baseline for assessing the accuracy of different prediction approaches under symmetric excitation conditions. The beamforming results obtained from these methods are summarized in Table 1.

**Table 1:** Beamforming performance comparison for single-beam radiation at *θ* = 0°.

Method	Peak Gain (dBi)	Sidelobe Level (dB)	Total Simulation Time (min)	Memory Usage (GB)
Full-wave simulation	29.02	−34.96	~1.0 × 10^4^	>50
Array factor	30.10	−34.8	<1	<1
Conventional AEP	28.79	−35.32	~40	~8
Partition-based AEP	28.86	−34.56	~120	~12
Proposed FERPP	29.10	−35.27	~180	~16

The array factor method exhibits noticeable deviations in both the main-beam shape and sidelobe distribution due to the neglect of mutual coupling and edge effects. As indicated in Table 1, the peak gain predicted by the array factor method is about 1 dB higher than that obtained from the full-wave simulation, while the sidelobe level is also slightly elevated. This overestimation reflects the limited accuracy of the array factor approach for tightly coupled finite arrays.

The conventional AEP method improves the overall pattern prediction by incorporating coupling effects through a single reference element. As shown in Table 1, the predicted peak gain is 28.79 dBi, which is about 0.23 dB lower than the full-wave simulation result. However, since only one active element pattern is used to represent all array elements, noticeable discrepancies in peak gain and sidelobe distribution still remain compared with the full-wave result.

The partition-based AEP method further reduces these deviations by accounting for region-dependent element behavior. The predicted peak gain is 28.86 dBi, which is closer to the full-wave result of 29.02 dBi. Nevertheless, since each region is still represented by a single active element pattern, the spatial variation of the electromagnetic environment within the edge region cannot be fully captured, which leads to residual discrepancies in the predicted radiation pattern.

The radiation pattern predicted by the proposed FERPP method shows very good agreement with the full-wave simulation over the considered angular range. As summarized in Table 1, the predicted peak gain is 29.10 dBi, which differs from the full-wave simulation result of 29.02 dBi by less than 0.1 dB. The sidelobe level is also very close to the reference value, indicating that both the main-beam shape and sidelobe distribution are accurately captured.

Compared with the conventional AEP and partition-based AEP methods, the FERPP method achieves higher prediction accuracy by explicitly modeling both central and edge elements, thereby effectively capturing the spatial variation of the electromagnetic environment in the finite array.

In addition, the computational cost remains significantly lower than that of the full-wave simulation, as indicated by the reduced simulation time and memory usage in Table 1. As a result, the proposed method enables accurate radiation pattern prediction for the broadside single-beam case.

#### 4.2.2. Scanned Single-Beam Radiation (*θ* = 30°)

The radiation patterns for a scanned beam at *θ* = 30° are shown in Figure 7. Compared with the broadside single-beam case (*θ* = 0°), beam scanning introduces stronger asymmetry in the electromagnetic environment and increases the sensitivity of radiation pattern prediction to mutual coupling and finite-array boundary effects, making accurate radiation pattern prediction more challenging. The corresponding beamforming results are summarized in Table 2.

As shown in Figure 7, the array factor method exhibits beam distortion and sidelobe mismatch under beam scanning because it neglects mutual coupling and finite-array boundary effects. This behavior is also reflected in Table 2: the array factor method predicts a peak gain of 30.10 dBi, which is about 1.11 dB higher than the full-wave reference (28.99 dBi). Such an overestimation indicates that the simplified point-source model becomes less reliable when a large progressive phase gradient is applied across a tightly coupled array, since the scan-dependent coupling and impedance mismatch are not captured.

**Table 2:** Beamforming performance comparison for single-beam radiation at *θ* = 30°.

Method	Peak Gain (dBi)	Sidelobe Level (dB)	Total Simulation Time (min)	Memory Usage (GB)
Full-wave simulation	28.99	−35.22	~1.0 × 10^4^	>50
Array factor	30.10	−35.33	<1	<1
Conventional AEP	26.18	−36.38	~40	~8
Partition-based AEP	27.37	−39.26	~120	~12
Proposed FERPP	28.22	−35.35	~180	~16

The conventional AEP method partially compensates for coupling effects by introducing an active element pattern, leading to some improvement over the array factor result. However, under beam scanning its limitation becomes evident: the predicted peak gain drops to 26.18 dBi, which is approximately 2.81 dB lower than the full-wave reference. This significant degradation suggests that a single reference AEP extracted at the array center is insufficient to represent the position-dependent electromagnetic environment when the beam is steered away from broadside. In particular, beam scanning breaks the symmetry of the element interactions and enhances boundary-induced coupling variations, so the central-element AEP cannot adequately represent the position-dependent element responses across the finite array under beam scanning. This limitation is also reflected in the sidelobe region. As shown in Table 2, the predicted sidelobe level is −36.38 dB, deviating from the reference result of −35.22 dB, indicating that the conventional AEP representation cannot fully reproduce the actual sidelobe distribution under beam scanning.

By further accounting for edge-related variations, the partition-based AEP method provides partial improvement over the conventional AEP approach. As summarized in Table 2, the predicted peak gain increases to 27.37 dBi, reducing the gain error to about 1.62 dB relative to the full-wave result. Nevertheless, a noticeable discrepancy still remains. This is mainly because each region is still represented by a single active element pattern, whereas under beam scanning the coupling environment changes continuously along the array aperture and cannot be fully approximated by a coarse regional assignment. Consequently, residual errors persist in both the main-beam region and the sidelobe structure. In particular, the sidelobe level predicted by this method reaches −39.26 dB, which is significantly lower than the reference value. This over-suppression of sidelobes suggests that the simplified regional modeling tends to distort the sidelobe distribution when the scan-induced coupling variations are not accurately captured.

The proposed FERPP method shows the best overall consistency with the full-wave simulation among the compared fast prediction approaches. The predicted peak gain is 28.22 dBi, corresponding to an error of approximately 0.77 dB with respect to the reference value of 28.99 dBi. Compared with the conventional AEP and partition-based AEP methods, this reduced error indicates that explicitly modeling the central and edge elements is beneficial under beam scanning, where boundary effects are amplified.

Compared with the broadside case, the slightly larger deviation observed under beam scanning can be mainly attributed to the stronger asymmetry of the electromagnetic environment and the enhanced scan-dependent variation when the beam is steered away from broadside. In the proposed FERPP method, the central-region responses are approximated from a calibrated reference response with spatial phase compensation. Although this approximation remains effective under beam scanning, its associated error becomes slightly larger than that in the broadside case. Nevertheless, the proposed method still maintains better overall agreement with the full-wave simulation than the conventional AEP-based approaches, indicating that retaining position-dependent response modeling remains beneficial for the scanned-beam case.

In addition, the sidelobe distribution and beam pointing accuracy are well preserved. As shown in Table 2, the predicted sidelobe level (−35.35 dB) is very close to the full-wave reference (−35.22 dB), demonstrating that the proposed method can accurately reproduce both the main-beam and sidelobe characteristics. In addition, Table 2 shows that the FERPP method achieves this accuracy with a substantially reduced computational cost compared with the full-wave simulation, confirming its robustness and efficiency for scanned single-beam scenarios.

#### 4.2.3. Multi-Beam Radiation (*θ* = −30°, 0°, and 30°)

Figure 8 shows the radiation patterns predicted by the different methods for the equal-weight three-beam configuration, with simultaneous beams directed toward *θ* = −30°, 0°, and 30°. Compared with the single-beam cases, this scenario is generally more challenging because the simultaneous excitation of multiple beams increases inter-beam interaction and requires more accurate modeling of the array radiation response.

The array factor method fails to accurately reproduce the relative beam amplitudes and sidelobe structure in the multi-beam case. As indicated in Table 3, it overestimates the peak gain by about 1.1 dB (25.32 dBi versus 24.21 dBi) compared with the full-wave reference. This deviation is mainly caused by the neglect of mutual coupling and finite-array boundary effects, which become more pronounced when multiple beams are simultaneously formed and large phase gradients are applied across the array for the off-broadside beams. The sidelobe distribution in Figure 8 shows noticeable discrepancies compared with the full-wave result, indicating that the simplified model cannot reliably capture the sidelobe characteristics in the multi-beam scenario.

**Table 3:** Beamforming performance comparison for equal-weight three-beam radiation.

Method	Peak Gain (dBi)	Sidelobe Level (dB)	Total Simulation Time (min)	Memory Usage (GB)
Full-wave simulation	24.21	−34.75	~1.0 × 10^4^	>50
Array factor	25.32	−35.70	<1	<1
Conventional AEP	22.26	−36.79	~40	~8
Partition-based AEP	23.15	−39.98	~120	~12
Proposed FERPP	23.87	−35.06	~180	~16

The conventional AEP method improves the overall pattern shape by incorporating coupling effects through a single reference element. However, the multi-beam scenario highlights its intrinsic limitation: the predicted peak gain is 22.26 dBi, which is approximately 2 dB lower than the full-wave reference. This significant underestimation suggests that a single central-element AEP cannot represent the position-dependent coupling variations for all elements when multiple beams (especially the two scanned beams at ±30°) are simultaneously excited. Consequently, imbalances among the three beams and discrepancies in the sidelobe regions remain.

By introducing spatially varying element responses, the partition-based AEP method provides partial improvement over the conventional AEP approach. The predicted peak gain increases to 23.15 dBi, reducing the gain error to about 1.1 dB relative to the full-wave result. Nevertheless, the predicted sidelobe level (−39.98 dB) is significantly lower than the reference value (−34.75 dB), suggesting an over-suppression of sidelobes. This behavior implies that coarse regional assignment using a limited number of representative AEPs may distort the sidelobe structure in the multi-beam case, where the electromagnetic environment varies continuously across the array and the coupling characteristics are different for the three simultaneously formed beams.

The proposed FERPP method shows the best overall agreement with the full-wave simulation across all three beams. As summarized in Table 3, the predicted peak gain is 23.87 dBi, corresponding to an error of about 0.34 dB relative to the reference (24.21 dBi), while the predicted sidelobe level (−35.06 dB) is also close to the full-wave result. In addition, the relative beam amplitudes and angular locations are well reproduced in Figure 8, indicating that the combined modeling of central and edge elements effectively captures the spatial variation of the electromagnetic environment in the finite array even under simultaneous multi-beam excitation. These results demonstrate that the proposed method maintains stable and robust radiation pattern prediction capability for the equal-weight multi-beam scenario, while requiring substantially lower computational resources than full-wave simulation.

#### 4.2.4. Summary and Discussion

Overall, the proposed FERPP method significantly reduces the computational cost of radiation pattern prediction by combining a compact calibration array with independently simulated edge subarrays, while maintaining prediction accuracy close to that of full-wave simulation, as demonstrated in Section 4.2.1, Section 4.2.2 and Section 4.2.3. Compared with the partition-based AEP method, the FERPP method requires slightly more computational time because additional full-wave simulations are performed for individual edge elements to accurately capture the position-dependent electromagnetic environment. Nevertheless, the total computational cost remains far lower than that of full-wave simulation. Moreover, since the computational complexity of the proposed method does not scale directly with the total number of array elements, it is well suited for beamforming analysis of large-scale tightly coupled antenna arrays.

### 4.3. Limitations of the Proposed Method

The proposed FERPP method is developed and validated for large-scale linear antenna arrays. While the method effectively accounts for mutual coupling and edge effects in this configuration, it is currently implemented for linear array geometries only. Extending the method to planar or more complex arrays would require additional modeling strategies, such as two-dimensional calibration regions and more elaborate edge representations.

In addition, the current implementation assumes identical element types and uniform inter-element spacing along the array. Arrays with irregular geometries, nonuniform spacing, or heterogeneous elements may require modified calibration strategies or more localized modeling procedures to maintain reliable prediction performance.

A further limitation is that the selection of the convergence threshold and the resulting calibration size is still based on a practical engineering criterion, and may therefore depend on the specific array configuration and the desired trade-off between prediction accuracy and computational cost. Moreover, compared with simplified analytical methods, the proposed framework still requires multiple localized full-wave simulations and response-integration steps, which increases the implementation complexity. These aspects will be investigated in future work.

### 4.4. Future Perspectives

The proposed FERPP framework provides a flexible basis for efficient radiation pattern prediction in large-scale antenna arrays. With appropriate extensions, the method can be generalized to planar arrays by introducing two-dimensional calibration regions and corresponding edge modeling strategies. At the same time, the proposed method enables fast radiation pattern evaluation by significantly shortening the prediction time, making it well suited for beamforming analysis and synthesis in large-scale tightly coupled antenna arrays where repeated radiation pattern calculations are required. Experimental validation using a fabricated large-scale array prototype will be considered in future work to further assess the practical applicability of the proposed method.

## 5. Conclusions

This paper proposes a radiation pattern prediction method, termed FERPP, for large-scale, tightly coupled linear antenna arrays, aiming to address the prohibitive computational cost of conventional full-wave simulation. By adopting a region-based modeling strategy, the proposed method combines accurate modeling of edge elements with an efficient equivalent representation of the central array region, enabling reliable prediction of array radiation performance with substantially reduced computational effort.

The proposed FERPP method is validated using a 1024-element linear antenna array as a representative example. Simulation results demonstrate that the radiation patterns predicted by FERPP closely agree with full-wave simulation results for both single-beam and multi-beam beamforming scenarios, with good consistency in main-beam gain, beam direction, and sidelobe characteristics. At the same time, the total simulation time is reduced to about 1.8% of that required by full-wave simulation, together with a significant reduction in memory usage.

A key feature of the proposed FERPP method is that its computational complexity does not scale directly with the total number of array elements, which makes the approach well suited for large-scale antenna array analysis. Although a 1024-element linear array is used as a representative example in this work, the proposed method is not inherently limited to this array size and can be applied to significantly larger arrays without a proportional increase in computational cost. The proposed method is particularly suitable for iterative beam synthesis and optimization workflows, where repeated radiation pattern evaluations are required. As a result, the proposed framework provides an efficient and practical tool for beamforming analysis and radiation performance evaluation in large-scale, tightly coupled linear antenna arrays.

## Data Availability

Data are contained within the article.
